# Supplementary material for: Single-atom Ni-N4 provides a robust cellular NO sensor
Source: Nat Commun. 2020 Jun 24;11:3188. doi: 10.1038/s41467-020-17018-6 (PMC7314822; doi:10.1038/s41467-020-17018-6)
Supplement: Supplementary file 1 — Supplementary Information [file 41467_2020_17018_MOESM1_ESM.pdf]

Supporting Information

Single-Atom Ni-N_4 Provides A Robust Cellular NO Sensor

Ming Zhou et al.

Supplementary Figures

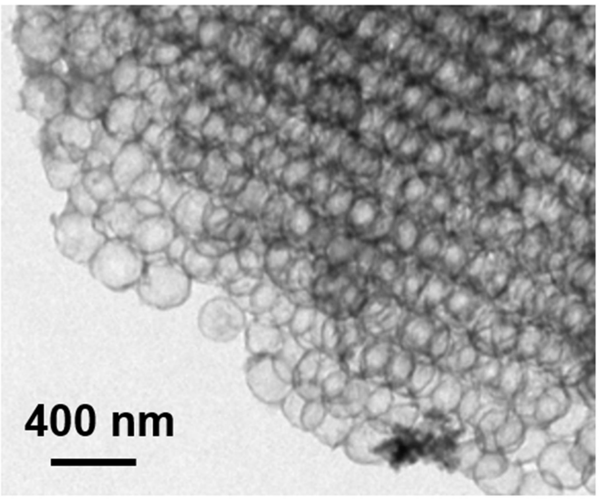

Supplementary Figure 1. TEM image of N-C materials. Scale bar: 400 nm.

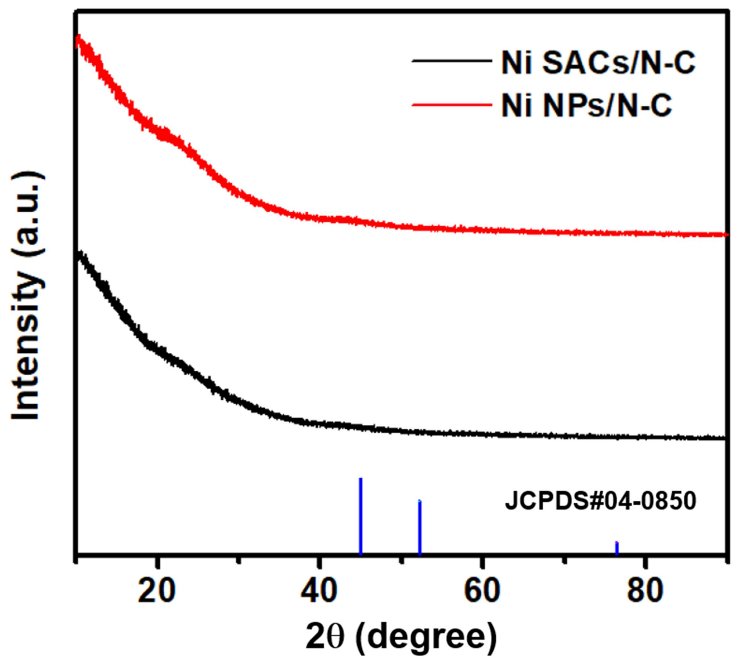

Supplementary Figure 2. XRD patterns of Ni SACs/N-C (black curve) and Ni NPs/N-C (red curve). The blue vertical line represents the standard Ni (JCPDS#04-0850).

Supplementary Note 1. In the XRD pattern of Ni NPs/N-C, no clear peak could be identified due to the low nickel content of 0.85% (wt%) of Ni in Ni NPs/N-C and the low resolution of XRD for the trace phase.

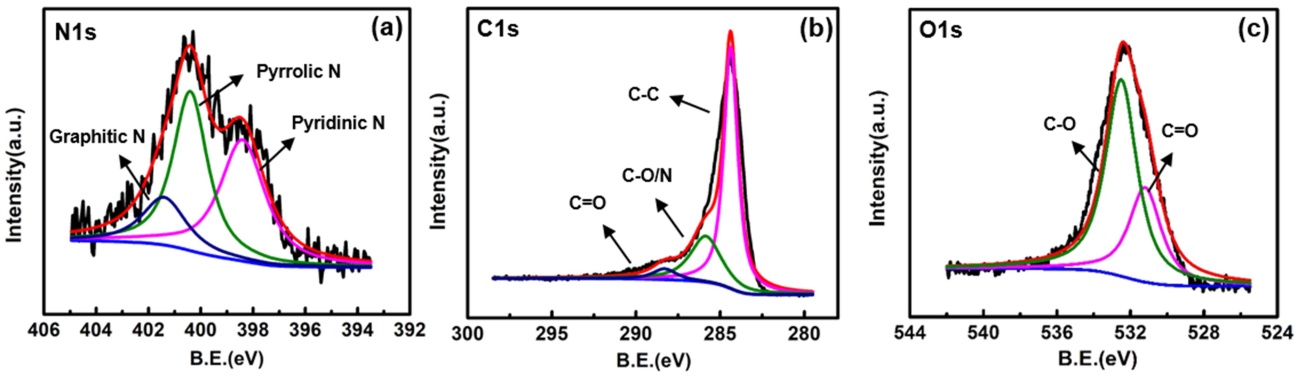

Supplementary Figure 3. XPS spectra for the (a) N 1s, (b) C1s and (c) O1s of Ni SACs/N-C.

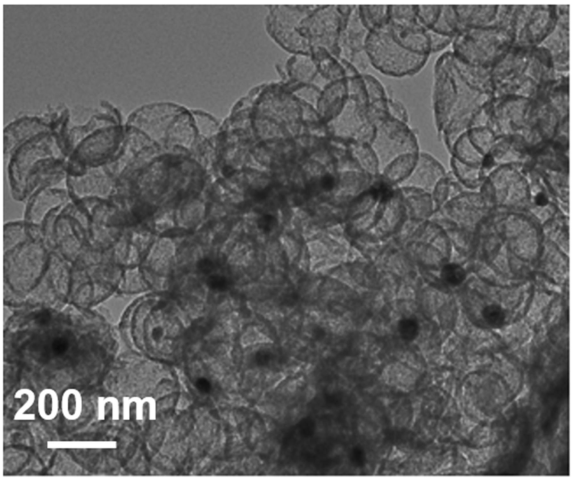

Supplementary Figure 4. TEM image of Ni NPs/N-C. Scale bar: 200 nm.

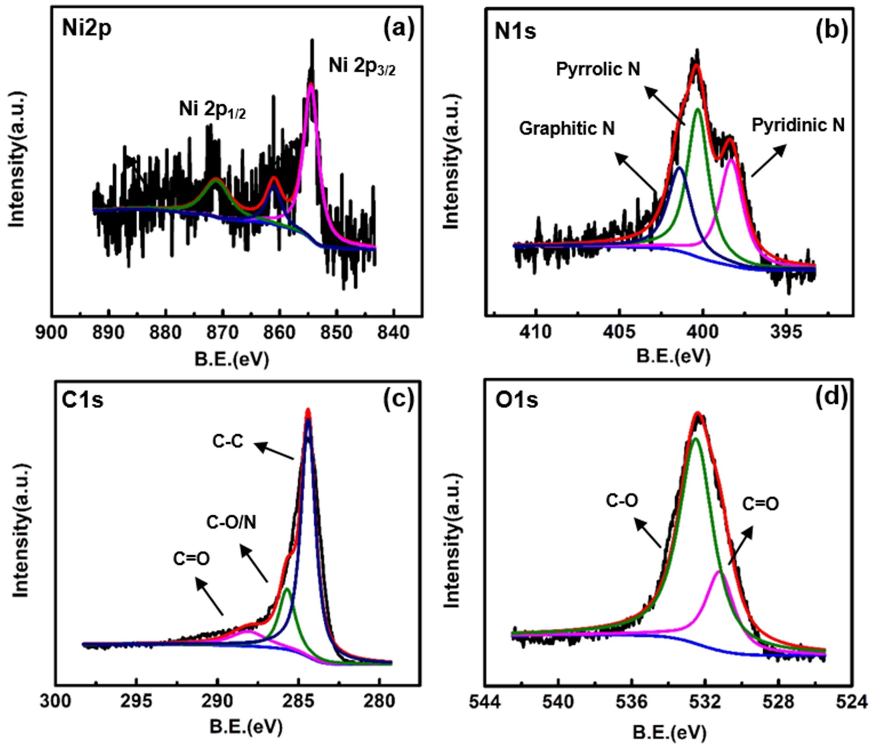

Supplementary Figure 5. XPS spectra for (a) Ni 2p, (b) N 1s, (c) C1s and (d) O1s of Ni NPs/N-C.

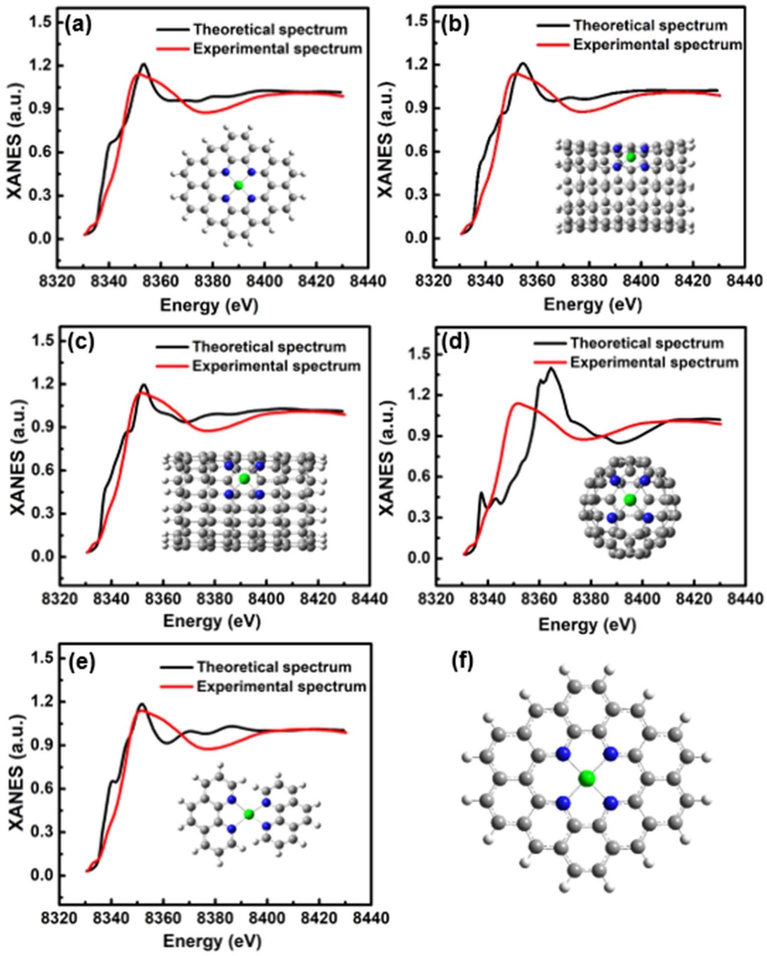

Supplementary Figure 6. Comparison between the XANES experimental spectrum for (a) graphene, (b) (6,6) carbon nanotubes, (c) (10,0) carbon nanotubes, (d) C_{60}, and (e) phenanthroline models of Ni SACs/N-C at Ni K edge (red lines) and the theoretical spectra calculated with the depicted structures (black lines). (f) The graphene model for the Ni-N_4 structure of Ni SACs/N-C.

Supplementary Discussion. To explore the geometrical and catalytic properties of the Ni SACs/N-C, several models with different curvatures were proposed for the Ni-N_4 structure. It can be seen that the graphene model best fits the experimental result. There are shoulder peaks around 8340 eV and 8350 eV. For the C_{60} model, the site and number of peaks are quite different from those in the experiment. For (6,6) and (10,0) carbon nanotubes, the shoulder peaks before 8350 eV are more than that in the experiment. As for the phenanthroline model, the difference from the experiment is the two flat peaks after 8360 eV. Theoretically calculated spectra in Supplementary Figure 6a-c and e are almost consistent with the experimental ones, indicating the models proposed with the carbon structures as the substrate for the Ni-N_4 structure may all exist, of which the most possible one is the graphene model.

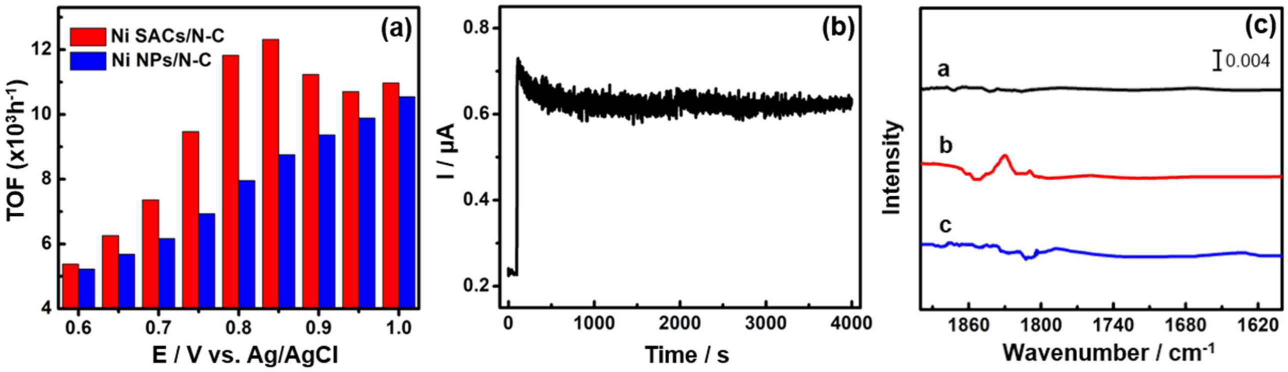

Supplementary Figure 7. (a) Turnover frequencies (TOFs) of Ni SAs/N-C and Ni NPs/N-C towards NO oxidation at different applied potentials. (b) Long-term durability of the Ni SACs/N-C based sensor towards 1 μM NO. (c) In situ FTIR spectra for the NO adsorption on Ni SACs/N-C. Spectrum taken immediately after a) NO admission, b) apply 0.8 V potential and c) remove +0.80 V potential on Ni SACs/N-C.

Supplementary Discussion. We investigated the NO adsorption behavior of the catalysts using Fourier-transform infrared (FTIR) spectroscopy to provide information about the dispersion and oxidation state of Ni. Supplementary Figure 7c shows that, when Ni SACs/N-C was exposed to NO solution, no infrared absorption signal was detected. After a potential of +0.80 V (*vs*. Ag/AgCl) was applied on the Ni SACs/N-C, an obvious absorption peak located at 1829-1842 cm^{-1} was observed (curve b), which was attributed to the frequency range of top adsorption NO on Ni site, according to the previous reports [1, 2]. At 1650-1600 cm^{-1}, there is no bridged adsorption peak of NO on Ni-Ni structure, which suggest that there are no adjacent Ni atoms in Ni SACs/N-C[2, 3, 4]. These results are consistent with our AC-STEM results in Figure 1 that Ni exists as an isolated single atom. Accordingly, when the 0.8 V potential was released, the adsorption signal disappeared on the curve c. The similar behavior of curve a and c before and after applying 0.8 V potential can indirectly account for SACs/N-C maintain the state of the single atom after the NO adsorption, which is beneficial for catalytic stability.

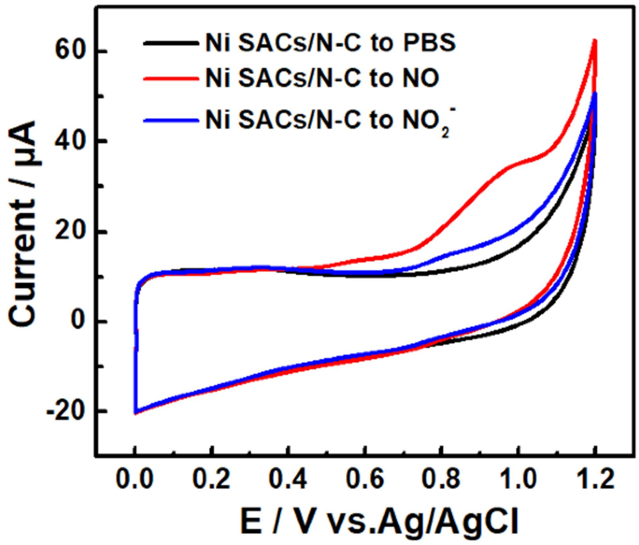

Supplementary Figure 8. CVs of Ni SACs/N-C electrodes in the absence (black line) and presence of 180 μM NO (red line) as well as NO_2^- (blue line) in deaerated PBS.

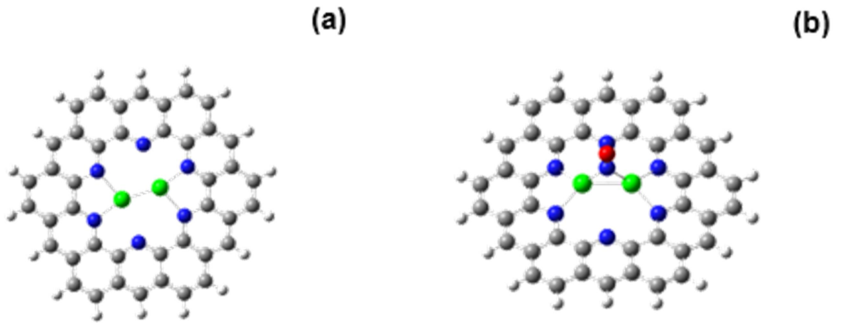

Supplementary Figure 9. (a) graphene-Ni_2 model, and (b) graphene-Ni_2 model with a NO molecule adsorbed of the Ni NPs/N-C.

Supplementary Discussion. As shown in Supplementary Figure 9a, the two Ni atoms have a bond between each other to model the situation of non-single atom catalysis. No imaginary frequency was also found in this case. The Ni NPs/N-C model with a NO molecule adsorbed is shown in Supplementary Figure 9b. The Gibbs free energy changes of the Equation (1) and (2) are -1.18 and 1.72 eV, respectively. The adsorption of the NO molecule is much more favorable for the Ni NPs than that for the Ni SACs/N-C. Thus the Gibbs free energy changes of the Equation (2) is quite large for the Ni NPs/N-C. The value 1.72 eV limits the catalytic reaction.

Supplementary Discussion.

More details of the proposed reactions on Ni SACs/N-C:

According to the mechanism reported previously [5], the electrocatalytic reaction could be described as follows.

$* + NO = *NO$ (1)

$*NO = *NO^+ + e^-$ (2)

$H_2O = H^+ + OH^-$ (3)

$*NO^+ + OH^- = *HNO_2$ (4)

$*HNO_2 = * + HNO_2$ (5)

The Gibbs free energy changes in the Supplementary Equation (1), (4) and (5) are 0.67, -6.06 and -0.99 eV, respectively, while the Gibbs free energy changes of Supplementary Equation (2) and (3) cannot be correctly calculated by the theoretical method employed here because the energies of the isolated particles such as e^- and H^+ are defined as zero in the quantum chemistry method. However, this can be solved through the equilibrium of the reaction $1/2\ H_2 = H^+ + e^-$ in electrochemical method.

Transition states in Supplementary Equation (1), (3), (4) and (5) are not found in our calculations. Although this may be caused by the DFT method used here, it can be speculated that there should not be high energy transition states for the weak adsorption in Supplementary Equation (1) and (5). If there is a transition state, the energy barrier is close to the energy change of the reaction. Since the Gibbs free energy changes of the Supplementary Equation (4) and (5) are minus, the rate-limiting step should be Supplementary Equation (2) and (3) in the total reaction: $*NO + H_2O = * + HNO_2 + H^+ + e^-$.

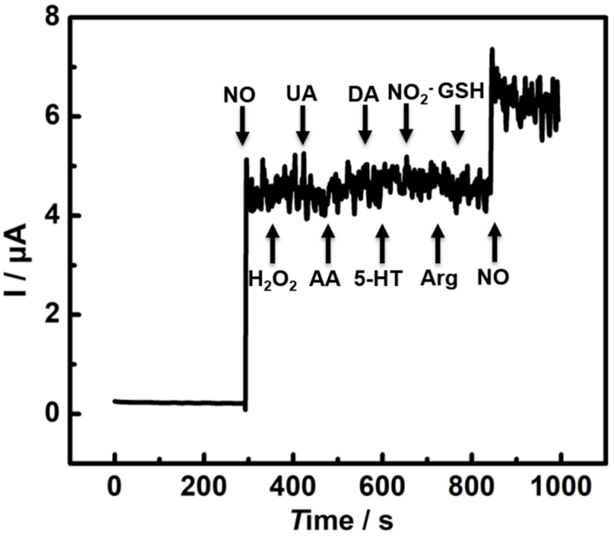

Supplementary Figure 10. Current responses of Ni SACs/N-C based sensor toward the successive addition of NO (10 μM), H_2O_2 (10 μM), UA (10 μM), AA (10 μM), DA (10 μM), 5-HT (10 μM), NO_2^- (10 μM), Arg (10 μM), GSH (10 μM) and NO (5 μM).

Supplementary Note 2. The selectivity of Ni SACs/N-C based sensor toward NO oxidation was studied with common interfering species in a biological system. The result demonstrates that the sensor produces a remarkably larger current response toward NO oxidation (10 μM) as compared with those toward interfering species with the same concentration of 10 μM.

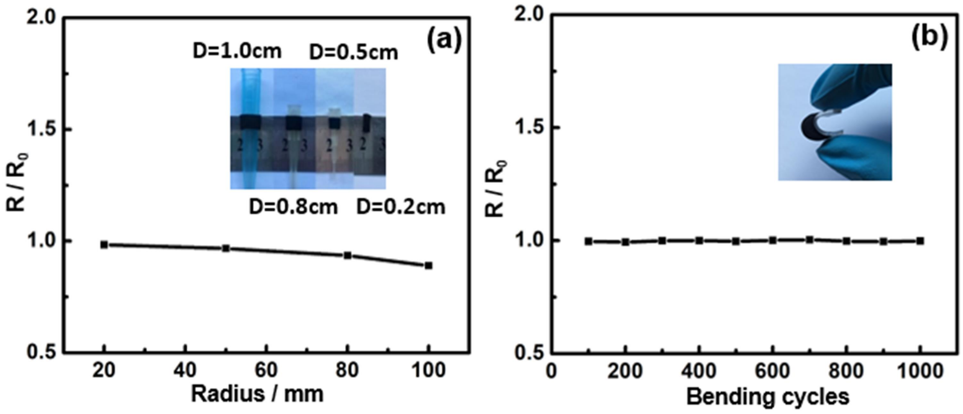

Supplementary Figure 11. (a) Relative variations of the ohmic resistance of the same Ni SACs/N-C/PDMS film electrode (length is 1.5 cm, width is 0.5 cm) along the number of bending to different curvatures and (b) bending cycles with a bending diameter of 1 cm. The bending is along the length direction. (R_0: original resistance; R: deformed resistance)

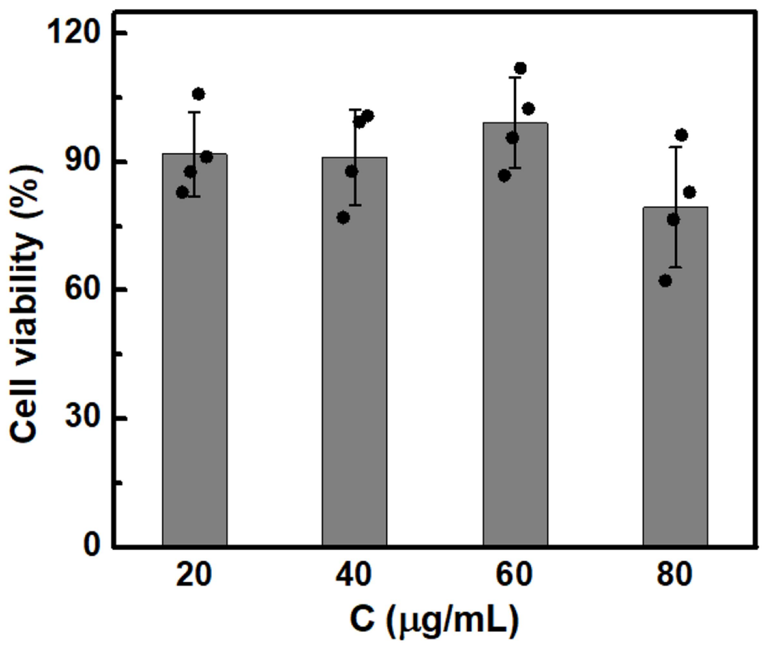

Supplementary Figure 12. CCK-8 assay of the viability of HUVECs cells treated with Ni SACs/N-C for 48 hours with different doses. Error bars=standard deviation (n=4).

Supplementary Note 3. The biocompatibility of the Ni SACs/N-C was investigated using human umbilical vein endothelial cells (HUVECs) by measuring cell viability with a Cell counting kit-8 (CCK-8) assay. The results show that the cell viability is basically above 80% after the cells were co-cultured with different concentrations of Ni SACs/N-C for 48 hours, implying that Ni SACs/N-C has minimal cytotoxicity and could thus be potentially used in biological studies.

Supplementary Table 1. Structural parameters extracted from the Ni K-edge EXAFS fitting (S_0^2=0.85)

Sample	Scattering pair	CN	R(Å)	$\sigma^2(10^3Å^2)$	ΔE_0(eV)	R factor
Ni-SACs/N-C	Ni-N/C	4.2	1.92	6.5	2.0	0.006

Supplementary Note 4. S_0^2 is the amplitude reduction factor; CN is the coordination number; *R* is interatomic distance (the bond length between central atoms and surrounding coordination atoms); σ^2 is Debye-Waller factor (a measure of thermal and static disorder in absorber-scatterer distances); ΔE_0 is edge-energy shift (the difference between the zero kinetic energy value of the sample and that of the theoretical model). *R* factor is used to value the goodness of the fitting. Error bounds that characterize the structural parameters obtained by EXAFS spectroscopy were estimated as N ± 20%; R ± 1%; σ^2 ± 20%; ΔE_0 ± 20%.

Supplementary Table 2. The optimized bond parameters of $(Ni\text{-}N_4)\cdots N$ geometric structure

Sample	Bond	Bond length(Å)	Bond	Bond length(Å)
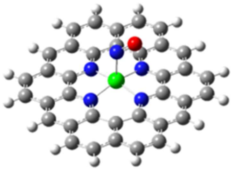 Ni SACs/N-C	(Ni-N)-NO	2.09	Ni-N	1.89
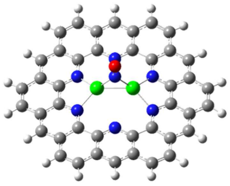 Ni NPs/N-C	(Ni-N)-NO	1.83	$Ni\text{-}N_1$	1.93
			$Ni\text{-}N_2$	2.08
			$Ni\text{-}N_3$	2.56

Supplementary Discussion. Ni atom in Ni SACs/N-C material is four-coordinated and has a $3d^8 4s^2$ electronic configuration, in which the coordination filling in all the 3d and 4p orbitals of Ni atom, making the NO adsorption is quite unfavorable. If NO absorbs on the Ni SACs/N-C, the bond length between the Ni and N atom of the NO molecule will be 2.09 Å, which is longer than that of Ni-N bond (1.89 Å) in the Ni SACs/N-C, i.e. the absorbed NO is unstable. In the Ni NPs/N-C, two Ni-N bonds have shorter bond lengths (1.93 and 2.08 Å) while the third Ni-N bond has a relative long bond length (2.56 Å). Thus, the number of coordinated N atoms decreases in Ni NPs/N-C and there is more unoccupied orbital in the Ni atoms. These make the adsorption of a NO molecule, i.e. the step (1) in the pathway from NO to HNO_2 become favorable accompanied by the bond length between the Ni and N atom of the NO molecule to be 1.83 Å. Accordingly, the following steps in the pathway of Ni NPs/N-C become less favorable because the Gibbs free energy change of the total reaction is fixed. Conversely, the unstable adsorption of a NO molecule on the Ni SACs/N-C reasonably explains the total catalytic activity of Ni SACs/N-C is higher than Ni NPs/N-C.

Supplementary Table 3. Comparisons of electrochemical parameters with some nanomaterial-based electrochemical NO sensors reported in the literatures

Sensing Interfaces	Applied Potentials (V vs Ag/AgCl)	Sensitivity ($nA \cdot uM^{-1} \cdot cm^{-2}$)	Detection Limit (nM)	Reference
**Ni SACs/N-C/PDMS**	+0.8	430.6	1.8	This work
**N-G/FePc/Nafion/PLL**	+0.945	210	180	[6]
**Au NTs/TiO_2 NWs/ Au NTs/PDMS**	+0.8	100	2.2	[7]
**GC/(S-G)-AuNP**	-	21.05	9.0	[8]
**Au NTs/PDMS**	+0.85	5.0×10^{3}	3.0	[9]
**GC/poly(PBZ)-AuNP**	+1.0	6.45×10^{-3}	3.7	[10]
**PtNPs**	+0.85	4.14	50	[11]
**MWCNTs**	+0.82	-	20	[12]

Supplementary Reference:

[1] Yu, M. *et al.* High density of end-oxygens induced by NO adsorption on (2×1) Ni-O/Ni (110) surfaces. *J. Phys. Chem. C* **123**, 21588-21592 (2019).

[2] Schenk, A, *et al.* Adsorption of NO and H, O/NO coadsorption at Ni (110) surfaces. *J. Vac. Sci. Techenol. A* **7**, 1996-2000 (1989).

[3] Qiao, B. *et al.* Single-atom catalysis of CO oxidation using Pt_1/FeO_x. *Nat. Chem.* **3**, 634-641 (2011).

[4] Hadjiivanov, K. *et al.* Use of overtones and combination modes for the identification of surface NO_x anionic species by IR spectroscopy. *Catal. Lett.* **68**,157-161 (2000).

[5] Brown, M. D. *et al.* Electrochemical nitric oxide sensors: principles of design and characterization. *Chem. Rev.* **119**, 11551-11575 (2019).

[6] Xu, H. Y. *et al.* Iron phthalocyanine decorated nitrogen-doped graphene biosensing platform for real-time detection of nitric oxide released from living cells. *Anal. Chem.* **90**, 4438-4444 (2018).

[7] Wang, Y. W. *et al.* Stretchable and photocatalytically renewable electrochemical sensor based on sandwich nanonetworks for real-time monitoring of cells. *Anal. Chem.* **90**, 5977-5981 (2018).

[8] Bhat, S. A. *et al.* Self-assembled AuNPs on sulphur-doped graphene: A dual and highly efficient electrochemical sensor for nitrite (NO_2^-) and nitric oxide (NO). *New J. Chem.* **41**, 8347-8358 (2017).

[9] Liu, Y. L. *et al.* Stretchable electrochemical sensor for real-time monitoring of cells and tissues. *Angew. Chem. Int. Ed.* **55**, 4537-4541 (2016).

[10] Vinu Mohan, A. M. *et al.* Electrochemical codeposition of gold particle-poly(2-(2-pyridyl) benzimidazole) hybrid film on glassy carbon electrode for the electrocatalytic oxidation of nitric oxide. *Sens. Actuators, B* **196**, 406-412 (2014).

[11] Zheng, D. Y. *et al.* Sensing of nitric oxide using a glassy carbon electrode modified with an electrocatalytic film composed of dihexadecyl hydrogen phosphate, platinum nanoparticles, and acetylene black. *Microchim. Acta* **176**, 49-55 (2012).

[12] Wang, Y. Z. *et al.* A multiwall carbon nanotubes film modified carbon fiber ultramicroelectrode for the determination of nitric oxide radical in liver mitochondria. *Bioelectrochemistry* **65**, 135-142 (2005).
